# Effect of Intra-Operative Topical 5-Fluorouracil Post-Functional Endoscopic Sinus Surgery: A Randomized Controlled Trial 

**DOI:** 10.22038/ijorl.2025.80571.3709

**Published:** 2025

**Authors:** Lekhaa Mohanraj, Somu Lakshmanan, Urvashi Singh

**Affiliations:** 1 *Senior Registrar, ENT & Head and Neck Surgery, Madras ENT Research Foundation (MERF), RA Puram, Chennai, India.*; 2 *Department of ENT and Head and Neck Surgery,Sri Ramachandra Medical College and Research Institure,Chennai, India.*; 3 *Department of ENT and Head and Neck Surgery, Apollo Hospitals, Chennai, India.*

**Keywords:** Randomized Control Trial, Functional Endoscopic Sinus Surgery, Outcomes, 5-Fluorouracil, Adhesions, Synechia

## Abstract

**Introduction:**

Endoscopic nasal surgery has numerous potential adverse effects, adhesions are at the top of the list. 5-fluorouracil (5-FU), which is an analogue of pyrimidine, is utilized in a wide variety of areas for the purpose of preventing adhesions. In the present investigation, our purpose was to analyze the impact of intra-operative application of 5-flurouracil in the nasal cavity following FESS, as well as to evaluate both subjective and objective outcomes.

**Materials and Methods:**

Following the acquisition of institutional ethical approval, a testing procedure that was randomized, prospective, and double-blinded was carried out. After the FESS, a cotton swab soaked in 1 mL of 5-flurouracil at 5 mg/mL was inserted in one side of the nasal cavity and a saline-soaked one in the other. Both of these swabs were kept in place for a period of five minutes. Postoperatively, patients were assessed over 6 months duration, wherein, adhesions, discharge, crustation, edema and polypoidal changes were analyzed. Subjective symptoms such as nasal block, nasal discharge and loss of smell were also assessed.

**Results:**

At the one-week follow-up, the test group had a significantly higher incidence of adhesions (32% vs. 11.76%, respectively) (p = 0.004) than the control group. Mucosal edema, crusting, polypoidal change and nasal discharge continued to significantly diminish in both groups. Furthermore, improvement in smell perception in the test group at the 20th week post-operatively was statistically significant (p= 0.014).

**Conclusions:**

Adhesions are less common in the early postoperative period in individuals who underwent FESS with or without polypectomy when 5-FU is administered topically. There is also an improvement in smell perception with a reduction of mucosal edema, crusting, polypoidal change and nasal discharge enabling better healing.

## Introduction

Inflammation of the nasal and paranasal sinus mucosa for at least twelve weeks is referred to as chronic rhinosinusitis (1). Three factors remain crucial for the normal physiological functioning of paranasal sinus: patency of the osteo-meatal unit (OMU), normal muco-ciliary transport and normal quality and quantity of secretions. With little tissue damage, nasal surgeries strive to cure disease, enhance nasal air flow, and enable sinus drainage by removing obstructions. 

Trauma to tissues can happen either directly during surgery or indirectly as a result of aberrant healing processes, including the formation of adhesions following the procedure (2). Adhesions represent one of the most frequent complications following endoscopic nasal procedures and are a reason for revision (3). As a means to reduce adhesions post-surgery, nasal splints, packing and spacers have been attempted (4-6). 

By blocking the enzyme thymidylate synthase, which is essential for DNA synthesis, the pyrimidine analogue 5-fluorouracil (5-FU) is able to reduce the proliferation of fibroblasts. Adhesions and difficulties connected to epithelial development can be effectively reduced using 5-FU, which is utilized by numerous medical specialties. For the treatment of actinic keratosis and psoriasis, its safety has been well-established in the fields of ophthalmology (7) and dermatology (8,9).

In this study, we examined the objective and subjective outcomes of utilizing topical 5-FU on nasal cavity post-functional endoscopic sinus surgery.

## Materials and Methods

From October 2021 through July 2023, our tertiary care teaching hospital in southern India conducted a randomized, double-blind controlled experiment (CTRI/2021/03/032286) in the department of ENT and Head and Neck Surgery. 

### Inclusion Criteria

1. Patients of either gender who are between the ages of 18 and 65 years 

2. Chronic rhinosinusitis with or without polyposis who have not responded to medication treatment 

3. Lund-Mackay Scoring difference of ≤2 between both sides in CT-PNS. 

EXCLUSION CRITERIA 

1. Revision endoscopic sinus surgery 

2. A difference of 3 or more in the Lund -Mackay scoring on both sides in CT-PNS. 

3. Patients with uncontrolled diabetes mellitus and hypertension 

4. Known allergy to 5-fluorouracil 

5. Pregnancy and lactation 

6. Pre-existing septal perforation or adhesion

### Study Design

Following the use of the Two Proportion Hypothesis testing method, the sample size was 68, with 80% power and 5% alpha error. We considered 'p' values below 0.05 statistically significant.

### Pre-operative Evaluation

A diagnosis of CRS was made based on the EPOS Criteria (10). Those enrolled in the study had no benefit from maximum medical therapy, which involved a trial of oral antibiotics and steroidal nasal sprays for 3 months. Pre - operative anaesthetic evaluation was done for all patients. A written informed consent was obtained. 

### Materials, set-up and technique

General anesthesia was used for the functional endoscopic sinus surgery. Pledgets soaked with oxymetazoline (0.05%) were used for pre-operative nasal packing. The surgical technique described by Messerklinger was followed for all patients. The surgery included an uncinectomy using a backbiter. 

**Fig 1:**
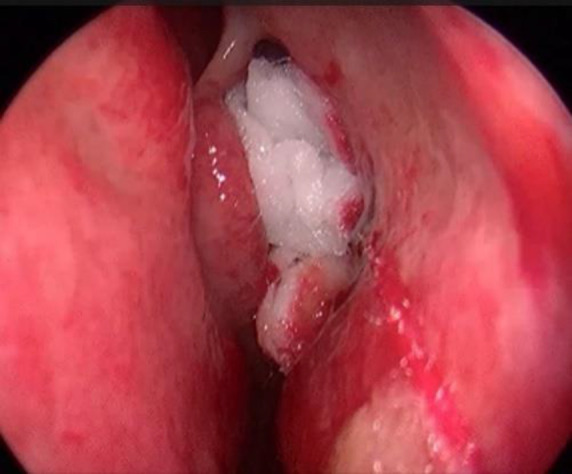
Endoscopic view of the left nasal cavity showing 5-FU-soaked cotton pledget placed in the middle meatus

The maxillary ostium was enlarged using a microdebrider. Anterior and posterior ethmoidectomy was done using a combination of instruments like curette, forceps and microdebrider. Ethmoidal bulla was removed using a curette or forceps to further expose the OMC. Ancillary procedures such as inferior turbinate submucous resection and septoplasty were performed when required. In patients with CRSwNP, sphenoidotomy and frontal sinusotomy was performed when indicated. Upon completion of surgery, application of topical 5 -FU was done unilaterally (Fig 1). 

A 0.9% saline solution was used to dilute 1 mL of 5-FU (1000 mg/20 mL. Under direct endoscopic visualization, a cotton pledget soaked in 1 ml of diluted 5-FU was inserted into one middle meatus, which was designated as the "test" nasal cavity. The 'control' group received a saline-soaked cotton pledget that was positioned contralaterally. A maximum of five minutes was allotted as contact time. The side selected for topical 5-FU application was randomized via random number generator. The content of both patties was kept unknown to the operating surgeon and the patient. A saline wash was given post-operatively. A non-absorbable nasal pack was placed post-procedure and removed after 24 hours.

### Discharge and follow-up

One day following surgery, patients were discharged from the hospital. A standardized post-operative strategy was implemented. They were discharged after removal of nasal pack, with oral Cefixime 200mg, twice a day, for a period of five days with anti-histamines and simple analgesics as per institutional protocol. For two weeks, a nasal spray containing a steroid (Mometasone 0.05%) was recommended. Saline nasal douching was to be done at home by the patients. At 1 week, 4 weeks and 20 weeks post-surgery, patients were seen in the outpatient department for follow-up appointments. Diagnostic nasal endoscopy was performed and the incidence of adhesions and synechiae in both the nasal cavities on endoscopy was documented. The degree of crusting, discharge and edema were classified based on Lund –Kennedy Endoscopic scoring system (11,12). A record was kept of any new complaints and the presence or absence of symptoms throughout the first week and the twentieth week of the study. The Nayak et al. study (13) was used to classify post-operative synechiae in the 20th week of follow-up endoscopy.

### Statistical Analysis

Group A was the "test" nasal cavity, and Group B was the "control" nasal cavity. The research used IBM SPSS Statistics for Windows 23.0. The Mann-Whitney U test was used to see if the independent group bivariate samples differed significantly. We used a paired analysis using a Chi-square test as we administered separate drugs to each nostril.

## Results

**Fig 2:**
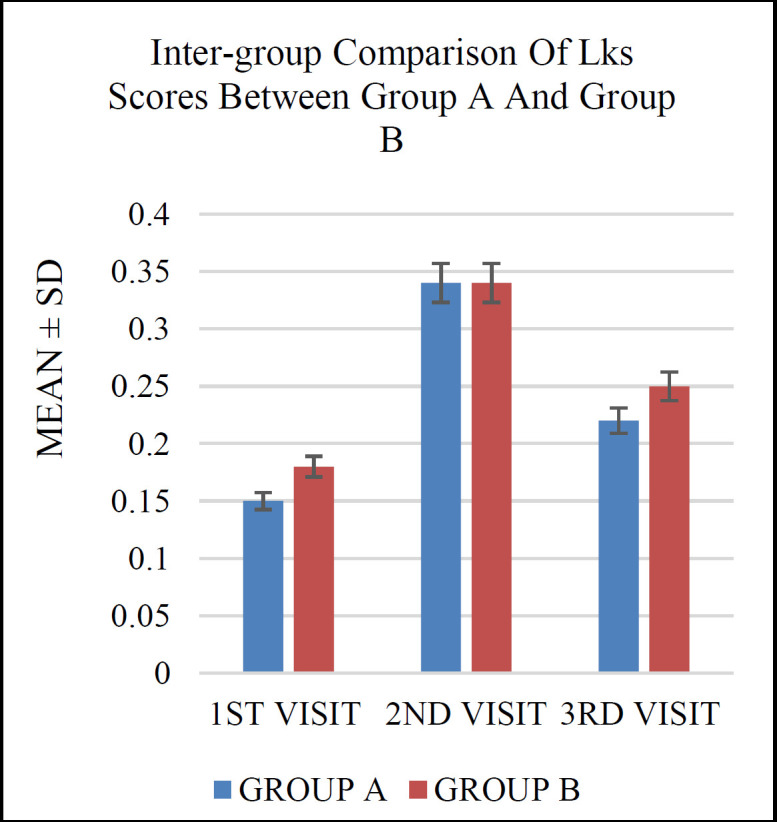
Bar diagram depicting comparison of Total LKS in Group A and Group B

68 patients were enrolled in the trial 38 (56%) were men and 30 (44%) were women. Participants averaged 42.51 years with a standard deviation of 12.5. On the first and twentieth weeks after surgery, Group A and Group B had statistically significant differences in Lund-Kennedy Scores (LKS). Group A had superior healing to compared to Group B as per endoscopic evaluation (Fig 2). 

Comparisons between Groups A and B were statistically significant during the first postoperative visit in week 1 (Table 1). 

Adhesions that persisted upto the 20th week post-operatively were termed synechiae.

**Fig 3:**
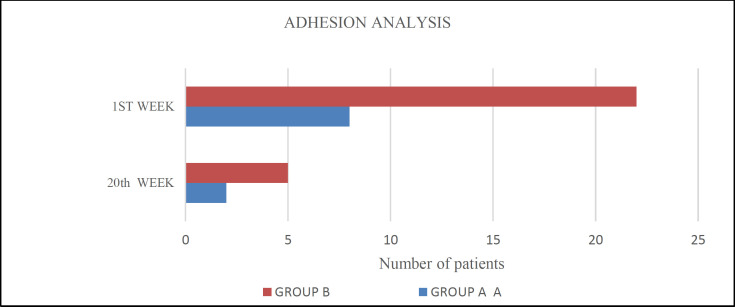
Bar chart showing analysis of intra-nasal adhesions at 1^st^ week and 20^th^ week.

At the 20th week post-operatively, 6 synechiae were noted in Group B and 2 were noted in Group A (Fig 3). 

50% of synechia were Type B as shown in Table 2. However, no significant difference in synechiae formation was noted between both groups (p=0.274).

A survey was used to analyze the subjective results. Nasal discharge, nasal block, and ability to detect odors were compared. No significant difference was noted in nasal discharge or nasal block. Smell perception was found to be statistically significant between Group A and Group B at the 20th week post-operatively (p value = 0.014).

## Discussion

Conventional wisdom holds that synechiae and adhesions within the sinonasal cavity are potential complications of functional endoscopic sinus surgery. Nasal procedures are often accompanied by the unexpected production of adhesions. A good surgical outcome is preceded by mucosal preservation and minimal distortion of surgical structures. Ineffective wound healing can result in significant scarring, the formation of synechiae and occlusion of the ostial passages. In their case series, Chambers et al. found that synechiae in the normal maxillary ostium and ethmoid cavity were associated with poor symptom outcomes (14). In a similar manner, Musy stated that synechiae were observed in more than half of the patients who underwent revision ESS (15). This was described in a series of seventy patients who underwent the procedure. Ramadan et al in their study of fifty-two individuals documented that over half had synechiae (16). 

Adhesions between the middle turbinate and the lateral wall are the root cause of most post-FESS failures, although there are ways to prevent them. Friedman et al. and Hewitt et al. (4,5) have detailed methods for medializing the middle turbinates. Nevertheless, this could lead to a loss of smell. Medialization by transseptal suturing lowers lateralization and synechia between the turbinate and lateral nasal wall, according to Bhalla et al (17). Injuring the nasal septum and middle turbinate can produce scarring, as noted by Bolger et al (18). 

Several types of meatal packing have been described over the years. There is no indication that one material is superior to another in preventing synechia, according to the meta-analysis by Weitzel et al (6). Endoscopically assisted cleaning eliminates crusts and early synechiae and protects the neo-ostia. A handful of studies have been conducted by using anti-metabolitic agents for preventing intranasal adhesions after endoscopic surgery. Three authors have recently investigated Mitomycin-C's capacity to stop adhesions following sinus surgery. Fifty-five participants participated in a double-blind RCT conducted by Chung et al. They administered mitomycin-C (0.4 mg/mL) to one side of the surgically treated middle meatus and saline to the other for five minutes. With Mitomycin-C, there was a tendency towards less adhesions, however this was not significant in statistical terms (19). 

In a 29-patient double-blind RCT, Anand et al. administered a saline pledget containing 0.5 mg/mL of Mitomycin-C for a duration of 5 minutes. No difference was noticed(20). In a second double-blind RCT, 38 patients received 0.5 mg/mL Mitomycin-C in the frontal os and saline on the other side. The authors discovered that the results for each side were identical (21). Similarly, the use of 5-FU for the same purpose has been studied here. 

By blocking the enzyme thymidylate synthase, which is essential for DNA synthesis, the pyrimidine analogue 5-FU is able to reduce fibroblast proliferation. Research has shown that 5-FU significantly slows down the proliferation of fibroblasts (22). Dermatologists use intralesional 5-FU to treat psoriasis. Moahajan et al. used 0.1 mL/cm2 and 5% of the medication infiltrated under the lesion and reported no harmful effects (9). Actinic keratosis has also been treated with 5-FU, which was used in the form of a topical cream containing 5% formulation (8).

The Fluorouracil Filtering Surgery Study Group evaluated usage of subconjunctival 5-FU in trabeculectomy. After a 5-year follow-up, 5-FU dramatically improved trabeculectomy outcomes(23). Donoso et al. and Singh et al. used intra-operative 5-FU in trabeculectomy (24,25). Usage of 5-FU in Dacryocystorhino- stomy is also well established (18,26). 

In the discipline of otorhinolaryngology, 5-FU is a medication that is considered to be relatively recent. It has been described by Nuseir et al. in their randomised controlled trial (RCT) of 69 patients (27), with regard to its role in post-endoscopic inferior turbinoplasty. At the conclusion of the first week following surgery, it was discovered that the individuals in the 5-FU group had a considerably lower incidence of adhesions and crustations than those in the previous group. 

In our study 68 patients underwent FESS for a comparable disease process in both sides of the nasal cavity, using a standardized technique. In Group A (5-FU), adhesions and crustations decreased statistically in the first week after surgery. Group A had a lower incidence of postoperative endoscopic findings such edema, polypoidal mucosa and discharge. There was a statistically significant improvement in smell perception in Group A at the 20th week post-operatively (p= 0.014). There was no statistically significant difference in the other subjective outcomes. In our study, the same patient acted as both test and control. On the other hand, tissue response to trauma varies from person to person, andchronic rhinosinusitis has an underlying immunological etiology. Because of the significant influence that both of these factors have on the results of FESS, it was necessary to choose a single group of patients and conduct tests on each individual nasal cavity as separate "arms." This allowed for the elimination of any potentially confounding factors. Patients with pre-existing nasal adhesions or septal perforations were excluded. Due to the fact that the condition was comparable on both sides, the extent of the surgery was comparable. In order to get a comprehensive and representative outcome of topical 5-FU after FESS, several confounding factors were removed from the equation. 

Even though the study's preliminary findings are encouraging, there could be a number of reasons why 5-FU's ability to reduce adhesion formation did not achieve clinical significance in the late post-operative phase. This is a result of two things: first, the study sample was relatively small, and second, the incidence of post-operative adhesions may be too low to demonstrate a clear therapeutic impact. Both factors are explicable. The distribution strategy used during nasal surgery may also affect 5-FU therapy. Ophthalmologists have applied 0.5 mg/mL 5-FU to the ocular surface during glaucoma filtration surgery. This trial employed a similar concentration and application duration, however bleeding from neighboring mucosal surfaces may have diluted the 5-FU concentration. A higher dosage may be beneficial for sinonasal surgery. No side effects related to usage of topical 5 -FU was encountered in our study. This is attributable to the reasonable concentration of 5 - FU that was used. However, glaucoma, corneal ulcers, and pain are some of the side effects of adjunctive 5-FU application that have been documented in ophthalmology literature. 

This study's use of validated objective outcome metrics and prospective data collection are among its key strengths. The absence of long-term outcome measurements is the analyses' primary drawback.

**Table 1:** Comparison of adhesions between Group A and Group B at 1^st^ week and 20^th^

**1** ^ST^ ** WEEK POST-OP**					
**GROUP B**		**GROUP A**		**Ratio**	**p value**
**Number of adhesions**	**Percentage of adhesions**	**Number of adhesions**	**Percentage of adhesions**		
22	32%	8	11.76%	2.72	0.004
20TH WEEK POST-OP					
GROUP B		GROUP A		Ratio	p VALUE
Number of adhesions	Percentage of adhesions	Number of adhesions	Percentage of adhesions		
5	2.94%	2	7.35%	0.4	0.274

**Table 2:** Type of synechia noted in Group A and Group B

**GROUPS**	**Number Of Synechia**	**Type Of Synechia**
GROUP A	1 1	Type B Type A
GROUP B	3 2 1	Type B Type A Type D

## Conclusion

With the absence of significant adverse effects, we are able to assert that the application of topical 5-FU intraoperatively leads to an improvement in both the subjective and objective outcomes of FESS during the initial post-operative period. The effectiveness and safety of 5-FU in endoscopic sinus surgery can be better determined by a large-scale, multi-centric randomized control trial that compares greater dosages and repeated applications.
